# Association between modified same-intercostal chest tube placement and acute postoperative pain after uniportal VATS lobectomy: an overlap weighted retrospective cohort study

**DOI:** 10.3389/fonc.2026.1797512

**Published:** 2026-04-13

**Authors:** Kang Qi, Weisiyu Abraham Qin, Zhimao Chen, Xining Zhang, Jian Li, Gang Lin, Shijie Zhang

**Affiliations:** 1 Department of Thoracic Surgery, Peking University First Hospital, Beijing, China; 2 Center for Tobacco Control Research and Education, University of California, San Francisco, San Francisco, CA, United States; 3 Department of Pathology, Erasmus MC Cancer Institute, Erasmus University Medical Center, Rotterdam, Netherlands

**Keywords:** acute postoperative pain, chest tube drainage, intercostal nerve, overlap weighting, uniportal VATS lobectomy, video-assisted thoracoscopic surgery

## Abstract

**Background:**

Chest tube placement is a routine but critical component of uniportal video-assisted thoracoscopic surgery (VATS). The routine method places the chest tube directly through the working incision, which may exacerbate acute postoperative pain. Multiple modified placement strategies have been proposed, yet evidence evaluating their comparative effectiveness on pain outcomes remains limited. This study examined the association between a newly proposed modified same-intercostal chest tube placement and acute postoperative pain following uniportal VATS lobectomy.

**Methods:**

This retrospective cohort study included adult patients undergoing elective uniportal VATS lobectomy at a tertiary medical center in Beijing, China, from June 2021 to June 2023. Among 1,701 screened patients, 1,082 met inclusion criteria. The exposure was tunneled same-intercostal chest tube placement, which creates a short subcutaneous and intramuscular tunnel along the superior border of the lower rib without traversing the rib or accessing an adjacent intercostal space. The primary outcomes were moderate-to-severe pain at rest within the first 24 hours. Propensity score overlap weighting was applied to achieve baseline covariate balance. Weighted logistic regression estimated adjusted odds ratios (aORs) and 95% confidence intervals (CIs). Pragmatic care-adjusted sensitivity analyses incorporated both baseline covariates and postoperative covariates.

**Results:**

The modified technique was associated with a lower rate of moderate-to-severe pain (16.8% vs. 27.1%). In weighted multivariable logistic regression analyses, modified technique was associated with 48% lower odds of acute postoperative pain (adjusted odds ratio [aOR] = 0.52, 95% CI = 0.35-0.79, *p* = 0.002) and a 38% lower adjusted risk (risk ratio [RR] = 0.62, 95% CI: 0.43-0.81, *p* < 0.001).

**Conclusions:**

The modified tunneled chest tube placement technique may meaningfully improve acute postoperative pain after uniportal VATS.

## 1 Introduction

Thoracic surgery, whether performed through open thoracotomy or minimally invasive approaches, has long been recognized as one of the most painful surgical procedures. This is due to the extensive chest wall manipulation and intercostal nerve irritation (1–3). Randomized and comparative studies confirm that video-assisted thoracoscopic surgery (VATS) reduces pain and accelerates recovery compared with thoracotomy (4, 5). Uniportal VATS, also referred to as single-incision VATS, has extended these benefits by allowing complex pulmonary resections through a single small incision (6). Although this evolution has markedly reduced surgical trauma and accelerated recovery compared with conventional thoracotomy (3, 7–9), clinically meaningful acute postoperative pain remains common in the early postoperative period and continues to limit ambulation, impair pulmonary mechanics, prolong hospitalization, and increase opioid exposure (10, 11).

A major driver of this postoperative discomfort is the chest tube, which is routinely placed after lung resection to maintain pleural pressure balance, evacuate air or fluid, and allow early detection of bleeding or lymphatic leakage (12, 13). Chest tubes can irritate or damage the intercostal neurovascular bundle and adjacent soft tissues, which explains their disproportionate contribution to pain and activity limitation. Electrophysiologic and imaging studies have demonstrated intercostal nerve dysfunction following tube insertion, consistent with both nociceptive and neuropathic mechanisms (1, 14).

In addition, Enhanced Recovery After Surgery (ERAS) pathways for lung resection treated chest tube management as a key element in optimizing recovery after lung resection (10). Its protocols specifically address the number, size, suction strategy, and timing of chest tube removal, recognizing that even modest reductions in tube-related pain can enhance ambulation, promote effective coughing, and prevent pulmonary complications (10). Within this framework, multiple approaches have been explored, including the use of smaller bore drains in selected patients (15), digital suction systems that allow earlier and more confident removal (16), early transition to water seal in proper cases to reduce air-leakage (17), and selective reduction from two drains to one after anatomic lung resection to preserving pleural drainage and air leak monitoring while minimizing the nociceptive burden imposed by the tube itself and to accelerate recovery (18, 19).

More recently, attention has also expanded from the device or system-level of the tube itself to the improvement of the chest tube placement techniques. In routine uniportal VATS practice, the chest tube is commonly inserted directly through the working incision and passed through the intercostal space in a straight path. Although technically efficient, this approach positions the tube in close proximity to the intercostal neurovascular bundle and can contribute to both nociceptive and neuropathic components of acute postoperative pain and can make deep inspiration, coughing, and ambulation uncomfortable (1, 14). Given that acute postoperative pain is a critical determinant of both the quality and speed of recovery, refinement of chest tube placement technique represents a clinically rational and modifiable factor within ERAS protocols. Emerging research and previous technique reports suggest that modified chest tube placement methods are feasible and may improve drainage efficacy and cosmetic outcomes (20–22). However, comparative effectiveness studies focused specifically on acute postoperative pain remain scarce.

Within this context, we empirically developed a modified tunneled chest tube placement technique that creates a short subcutaneous and intramuscular tunnel within the same intercostal space which advances the drain along the superior border of the lower rib to minimize bundle contact, reduce skin-site traction, and improve tube stability during respiration and ambulation. This study retrospectively compared the modified tunneled intercostal chest tube placement and routine chest tube placement techniques after uniportal VATS to examine their associations with acute postoperative pain and evaluate whether the modified approach offers improved early postoperative comfort.

## 2 Method

### 2.1 Study design

Data for this retrospective cohort study was extracted from the electronic health records (EHR) of a tertiary medical center in Beijing, China. The dataset included 1,701 patients who underwent uniportal VATS lobectomy between June 2021 and June 2023. The study protocol was reviewed and approved by the Institutional Review Board (IRB) for Clinical Investigations at Peking University First Hospital, the Affiliated Hospital of Peking University Health Science Center (IRB No. 2022-673). Given the retrospective nature of the study and the use of de-identified data, the requirement for written informed consent was waived. The study was conducted following the ethical standards of the 1975 Declaration of Helsinki. Eligible participants met the following inclusion criteria: (1) age ≥ 18 years; (2) non-emergency uniportal VATS procedures; (3) preoperative clinical T1 N0–1 M0 lung cancer with a tumor < 3 cm in diameter; (4) surgical incision ≤ 3 cm in length. Exclusion criteria included: (1) previous history of malignancy, thoracic surgery, or receipt of preoperative neoadjuvant therapy; (2) pathological tumor (pT1) size > 3 cm in diameter; (3) surgical incision > 3 cm; (4) non-lobectomy procedures, such as wedge resection, segmentectomy, sleeve resection, or pneumonectomy; (5) occurrence of Clavien–Dindo grade III–V complications; or (6) incomplete perioperative medical records. Patients who developed severe postoperative complication (Clavien–Dindo grade III–V) was excluded given these events trigger substantial deviations in postoperative management, including prolonged chest tube duration and intensified analgesic use. This exclusion aimed to reduce heterogeneity in care trajectories and facilitate a clearer assessment of early resting pain.

**Figure 1:**
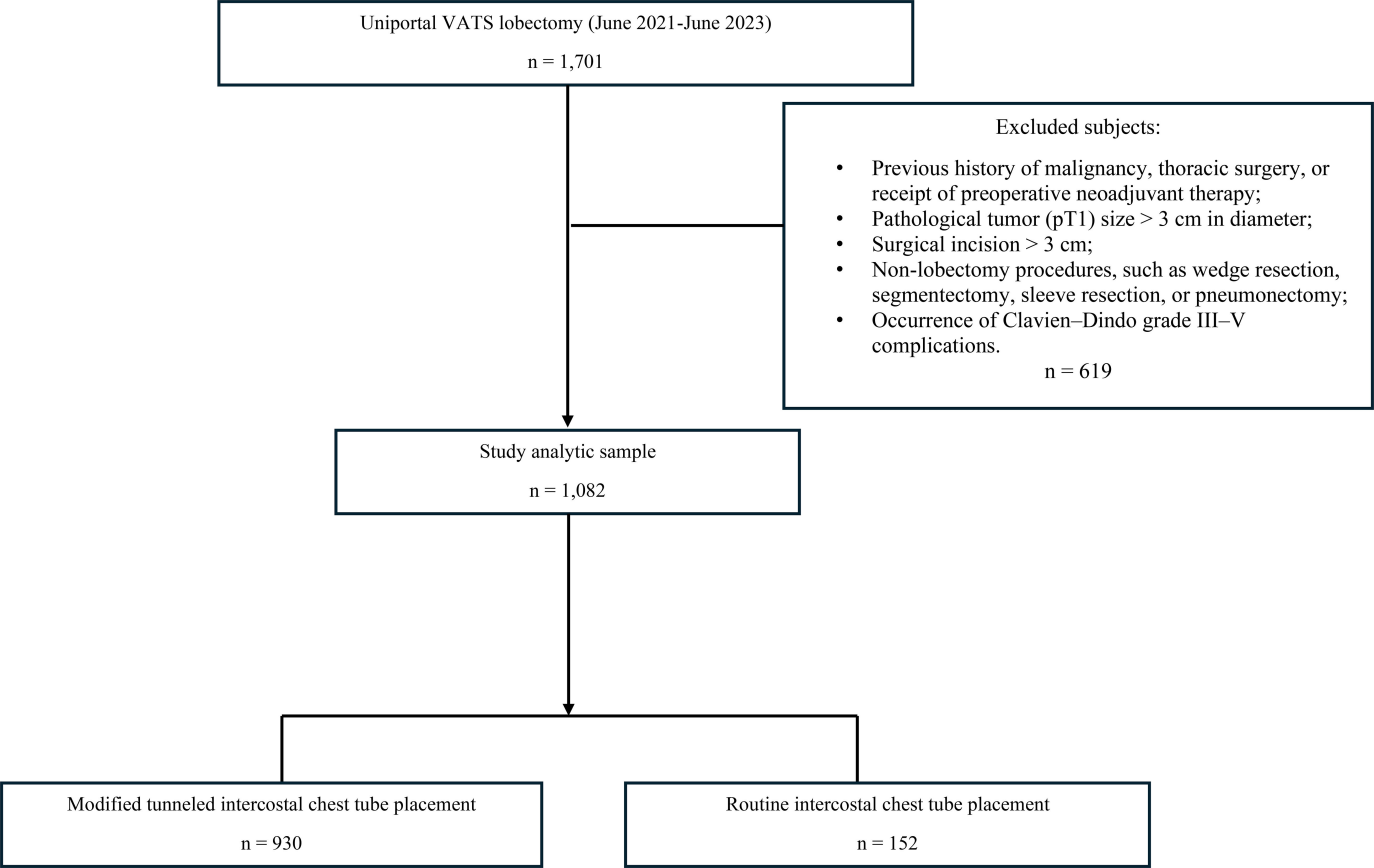
Study cohort sample construction flow diagram.

Among the 1,701 screened patients who received the uniportal VATS lobectomy, 1,082 patients who met the inclusion criteria composed the analytic sample of the current study. Among them, 930 (86%) received the modified tunneled intercostal chest tube placement and 152 (14%) received the routine chest tube placement (**Figure 1**). Assignment of modified versus routine chest tube placement was not based on pre-specified patient-level criteria. The modified tunneled chest tube placement technique was adopted as a technical refinement empirically and progressively adopted in routine practice across the surgical teams during the study period. During this time, the attending surgeon roster and operative teams remained stable and there were no institutional changes to the operative workflow or surgical equipment for uniportal VATS lobectomy. Operations in both groups were performed by the same surgical teams throughout the study window with similar workloads and comparable scheduling.

### 2.2 Operative procedures

Patients underwent uniportal VATS lobectomy in accordance with standard institutional practice guidelines. Perioperative analgesia during the study period followed established institutional protocols. Intraoperative opioids were used as part of routine general anesthesia management. Upon completion of incision closure, intercostal infiltration with 0.2% ropivacaine (5 mL per patient) was administered through intercostal injection. Postoperatively, patients routinely received patient-controlled analgesia (PCA) with sufentanil, and NSAIDs were used for postoperative analgesia as clinically indicated.

In the routine method of chest tube placement after pulmonary resection, the drainage tube is directly inserted through the same intercostal space as the uniportal incision and passes perpendicularly through the intercostal incision into the pleural cavity under direct thoracoscopic visualization. This direct insertion technique is routinely employed in uniportal video-assisted thoracoscopic surgeries, where it serves as the standard method for efficient drainage of pleural air and effusion. Compared with the routine method, the modified approach establishes a short subcutaneous tunnel within the same intercostal space and introduces the tube through the intercostal muscle along the superior border of the lower rib. This modified placement does not need to traverse the rib or cross into an adjacent intercostal space.

Specifically, before closing the incision, the chest tube insertion point was identified at the posterior margin of the original uniportal incision, approximately 2 cm posterior to the wound edge within the same intercostal space. No additional skin incision was made with only blunt dissection was required. A subcutaneous tunnel was then created by gently inserting curved hemostatic forceps through the subcutaneous tissue beneath the incision and advancing them posteriorly in parallel with the direction of the intercostal space, forming a tunnel approximately 2 cm in length. The dissection depth was limited to the intercostal muscle layer overlying the superior edge of the lower rib, avoiding the inferior margin of the upper rib to prevent injury to the intercostal neurovascular bundle.

**Figure 2:**
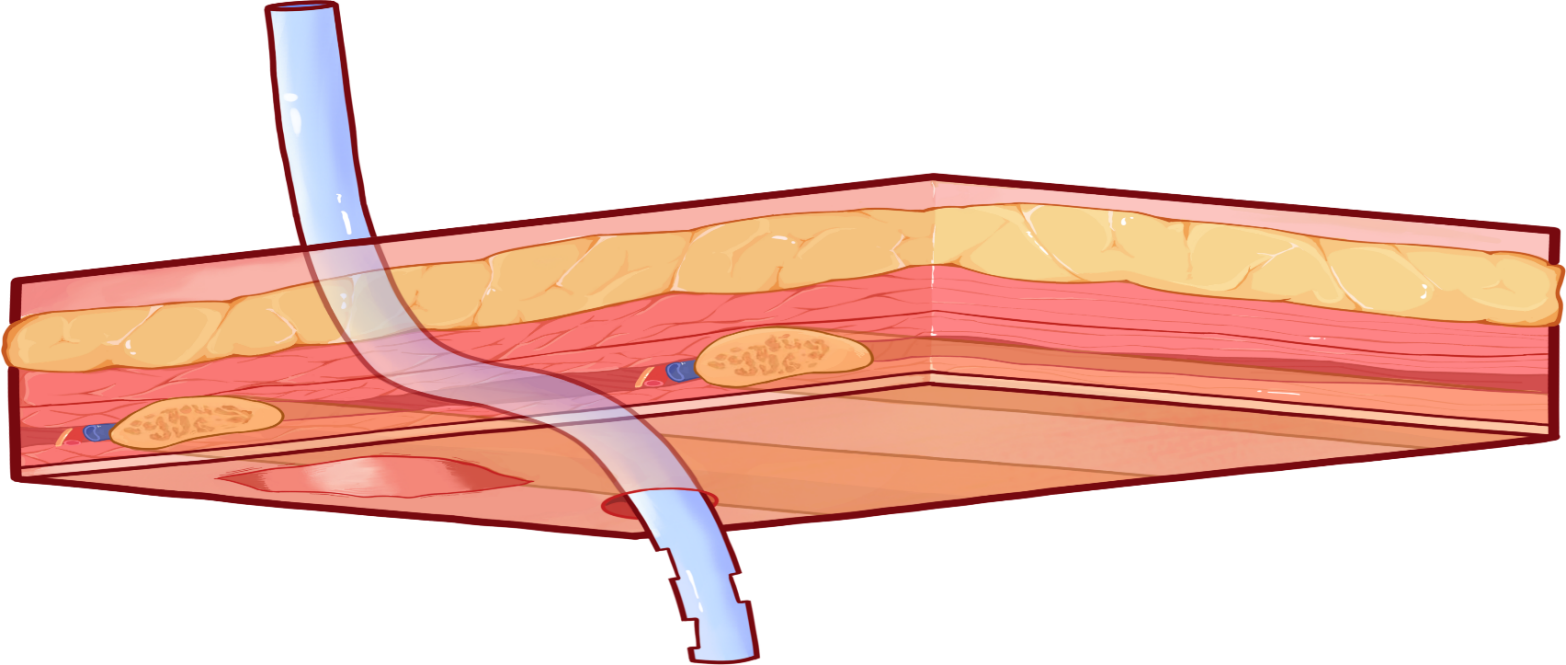
Internal chest-wall perspective view of the modified tunneled chest tube placement. This view demonstrates the tunnel trajectory through the intercostal space.

**Figure 3:**
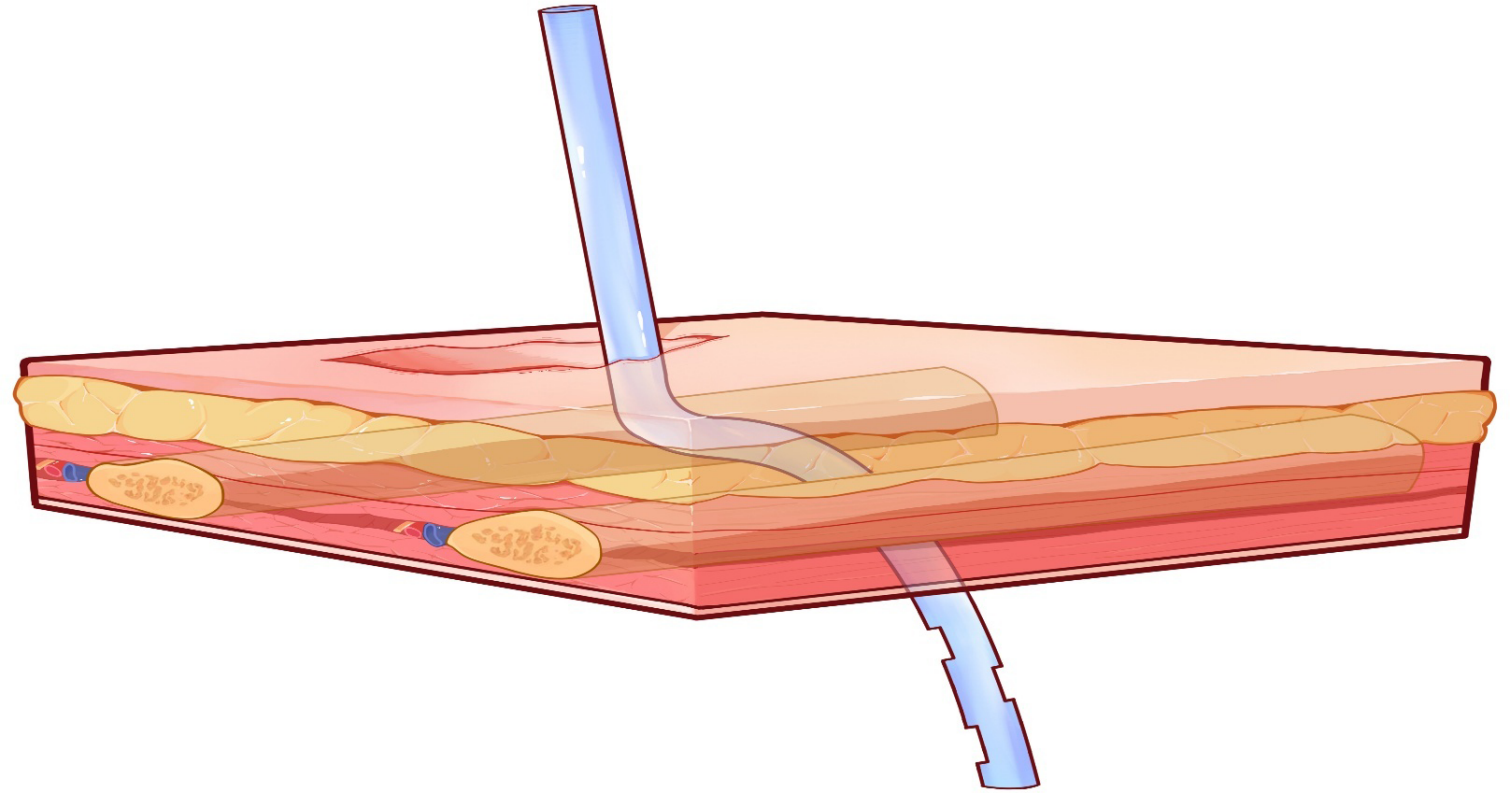
External chest-wall perspective view of the modified tunneled chest tube placement. This view demonstrates the skin incision and tube entrance site.

At the posterior end of the tunnel, pleural entry and tube insertion were performed perpendicularly through the intact intercostal muscle layer along the superior border of the lower rib, without crossing the lower or upper rib. Since this step involves separation through the intercostal tissues along an anatomic plane, excessive bleeding is uncommon. Hemostasis, when needed, was managed using standard intraoperative measures. The chest tube was then advanced through the subcutaneous tunnel from the incision site until its tip emerged from the posterior puncture point and entered the pleural cavity. Under thoracoscopic visualization, the tube position was adjusted so that the distal tip was directed toward the lung apex or the posterior basal region as needed. After confirming appropriate placement and patency, the tube was secured, and the incision was closed in layers. After confirming appropriate placement and patency, the tube was secured to the skin using non-absorbable sutures, consistent with the standard fixation method used for both the routine and modified techniques, and the dressing strategy followed the same institutional standard in both groups. **Figures 2** and **3** illustrate this modified placement technique, with **Figure 2** showing the internal chest-wall perspective and **Figure 3** showing the external chest-wall perspective.

Early postoperative chest tube management was standardized across both groups. Tubes were connected to a water-seal drainage system with suction routinely set at −5 to −10 cmH_2_O during the first 24 hours, after which water seal was used per standard practice. Chest tube removal criteria were consistent between groups and included absence of an air leak and drainage volume less than 200 mL over 24 hours. Routine chest X-ray examinations were conducted subsequently within 24 hours after surgery to verify the effectiveness of lung re-expansion status.

### 2.3 Study measures

*Exposure variable*. The exposure variable of the current study was the method of chest tube placement (modified tunneled intercostal vs. routine chest tube placement).

*Outcome variable*. The outcome variable was the acute postoperative pain at rest within the first 24 hours after surgery. Acute postoperative pain at rest was assessed as part of nursing care and documented in the medical record. To reflect usual care conditions, pain was recorded while patients were resting in bed rather than during mobilization. In routine practice, the resting NRS was obtained during morning nursing rounds on postoperative day 1, occurring within 24 hours after incision closure. Nursing staff responsible for pain documentation were not involved in chest tube placement and were not informed of the placement technique used. Pain intensity was assessed based on the numeric rating scale (NRS) and dichotomized into mild pain (NRS < 4) and moderate-to-severe pain (NRS ≥ 4) (23).

*Covariates.* The current study incorporates a range of baseline demographic and clinical covariates to adjust for factors that may confound the relationships. Demographic characteristics included age (dichotomized as <65 or ≥65 years at the time of operation) (23), biological sex (female/male), body mass index (BMI, kg/m²) categorized based on Chinese adult standards (24). As no participants had BMI<18.5 in the current analysis, BMI was categorized as three levels ([18.5–24.0), [24.0–28.0), ≥28.0). Educational level was categorized as less than college versus college or higher. Insurance type was classified as ensured under Beijing Urban Employee Basic Medical Insurance (UEBMI) or others (25). Since the implementation of China’s universal medical insurance system in 2016, all patients in this cohort were insured, although the extent of coverage and reimbursement rate varied across regions and insurance types.

Smoking history was coded dichotomously (yes/no), defined as any active tobacco use within 14 days before surgery. Patients are counseled to avoid all tobacco use for a minimum of two weeks before surgery. Chest tube size was categorized into three levels, based on the standard internal diameter of the tube measured in French units (#18, #20, and #24 French). Nonsteroidal anti-inflammatory drugs (NSAIDs) use within 14 days (2 weeks) prior to surgery was recorded as days of use to quantify preoperative analgesic exposure. Data on both oral and parenteral NSAID administration were extracted from electronic medical records, including prescriptions for chronic pain or inflammatory conditions. Tumor size was measured in millimeters (mm) based on pathological or radiographic assessment.

For the pragmatic care-adjusted sensitivity analyses, postoperative clinical variables were also incorporated as covariates, including drain duration (days), operation length (hours), and length of inpatient (days). Additional descriptive variables included forced expiratory volume in one second (FEV_1_) in liters, and number of lymph nodes examined. These variables were summarized to provide a more comprehensive characterization of the baseline profiles of the population in the current study.

### 2.4 Statistical analysis

For descriptive analysis, unweighted statistics were calculated to rigorously characterize the sample’s baseline demographic and clinical features, as well as the prevalence of outcome measures. Continuous variables were measured as means with standard deviations while categorical variables were summarized as counts with proportions. Between-group comparisons were performed using Welch’s t-test for continuous variables and Pearson chi-square tests for categorical variables.

Given the imbalance nature in baseline characteristics between patients receiving modified and routine chest tube placement, overlap weighting based on the propensity score was applied to mitigate confounding and enhance comparability between groups. This method focuses on the average effects in the “overlap population”, representing patients for whom either treatment is clinically plausible given their covariate profiles, thereby approximating the characteristics of a randomized clinical trial (26). This approach yields clinically interpretable, generalizable, and statistically robust estimates, while minimizing bias that may occur from other probability weighting or matching methods (27). Propensity scores were estimated using a logistic regression model predicting the probability of receiving the tunneled chest tube method, conditional on prespecified baseline covariates. Postoperative variables were excluded to prevent potential collider bias and preserve the temporal ordering between exposure and covariates. Following estimation of the propensity scores (PS), overlap weights were calculated, assigning weights of *(1 − PS)* to tunneled chest tube patients and *PS* to non-tunneled patients (26). Covariate balance was visually and numerically assessed using Love plots and propensity score density plots, confirming satisfactory overlap between groups (28, 29). For the current study, overlap weighting was applied to adjust for baseline covariates using propensity score modeling, incorporating age, biological sex, BMI category, highest education level, insurance type, smoking history, tube size, preoperative NSAIDs use, and tumor size.

The univariate logistic regression models were conducted with the overlap-weighted sample to estimate crude associations between chest tube placement method and acute postoperative pain outcomes, as well as between each covariate and acute postoperative pain outcomes. Subsequently, the multivariable logistic regression model was fitted within this weighted sample, adjusting for the same baseline covariates used in the propensity score model for robust estimation. In addition, absolute risk, risk differences, risk ratios were calculated with the weighted sample to provide bias-adjusted effect estimates.

For the pragmatic care–adjusted sensitivity analysis, postoperative covariates were incorporated to better reflect real-world clinical practice. The findings were interpreted as associational, as such postoperative covariates are downstream of exposure and may potentially act as mediators or colliders (30). Additionally, univariate and multinomial logistic regression analyses using unweighted data were conducted as complementary sensitivity analyses.

A complete-case approach was adopted due to the relative low proportions of missingness. All statistical analyses were conducted using R (version 4.5.1) with the R-Studio environment. Statistical significance of *p*-value was assessed two-sided with a threshold of alpha = 0.05.

## 3 Results

### 3.1 Descriptive statistics

The unweighted descriptive statistics of baseline demographic and clinical characteristics are demonstrated in **Table 1**. A significantly lower proportion of patients who received the modified placement reported acute postoperative pain (16.8%) compared with those who received the routine placement (27.1%; *p* = 0.004). Between group differences were also observed in insurance coverage and tumor size. A higher proportion of patients in the modified placement group were covered by the UEBMI insurance compared with the routine group (61.4% vs 48.0%; *p* = 0.028). In addition, patients who received the modified placement had a slightly larger mean tumor size (18.7 mm vs 17.3 mm; *p* = 0.028).

**Table 1:** Descriptive statistics of patients underwent uniportal video-assisted thoracic surgery (VATS) (n = 1,082).

Variables	Full sample	Modified placement	Routine placement	*p*-value
	n=1082	n=930	n=152	
ICT placement				
Routine	152 (14.0%)			
Modified	930 (86.0%)			
Acute postoperative pain intensity				0.004^**^
0 to 3	882 (81.5%)	771 (83.0%)	111 (73.0%)	
4 to 10	200 (18.5%)	159 (17.0%)	41 (27.0%)	
Age				0.108
< 65	677(62.6%)	573 (61.6%)	104 (68.4%)	
≥65	405(37.4%)	357 (38.4%)	48 (31.6%)	
Biological sex				0.918
Female	595 (55.0%)	512 (55.1%)	83 (54.6%)	
Male	487 (45.0%)	418 (44.9%)	69 (45.4%)	
BMI (kg/m^2^)				0.434
[18.5-24.0)	693 (64.0%)	591 (63.6%)	102 (67.1%)	
[24.0-28.0)	243 (22.5%)	215 (23.1%)	28 (18.4%)	
≥28.0	146 (13.5%)	124 (13.3%)	22 (14.5%)	
Highest education level				0.995
High school or lower	769 (71.7%)	661 (71.1%)	108 (71.1%)	
College or higher	313 (28.9%)	269 (28.9%)	44 (28.9%)	
Insurance type				0.028^*^
UEBMI	650 (60.1%)	571 (61.4%)	73 (48.0%)	
Others	432 (39.9%)	359 (38.6%)	79 (52.0%)	
Smoking history				0.686
No	752 (69.5%)	642 (69.0%)	110 (72.4%)	
Yes	330 (30.5%)	288 (31.0%)	42 (27.6%)	
Chest tube size				0.893
#18	206 (19.0%)	177 (19.0%)	29 (19.1%)	
#20	670 (61.9%)	578 (62.2%)	92 (60.5%)	
#24	206 (19.0%)	175 (18.8%)	31 (20.4%)	
NSAIDs use history (days)	3.7 ± 1.7	3.7 ± 1.7	3.5 ± 1.6	0.301
Tumor size (mm)	18.5 ± 7.3	18.7 ± 7.3	17.3 ± 7.1	0.028^*^
Number of lymph nodes	15.6 ± 6.2	15.7 ± 6.3	15.1 ± 5.9	0.257
FEV_1_ (liters)	2.5 ± 0.6	2.5 ± 0.6	2.5 ± 0.6	0.558
Drain duration (days)	3.9 ± 2.4	3.9 ± 2.4	3.8 ± 2.2	0.388
Operation length (hours)	2.8 ± 0.8	2.9 ± 0.9	2.8 ± 0.8	0.645
Length of inpatient (days)	5.1 ± 2.6	5.2 ± 2.7	5.0 ± 2.2	0.364

The descriptive measures presented in this table are all unweighted. Continuous variables are presented as mean ± standard deviation while categorical variables are presented as frequency (proportion). ICT, intercostal chest tube placement; FEV_1_, forced expiratory volume in 1 second measured by liters; NSAIDs, Nonsteroidal Anti-Inflammatory Drugs. Smoking history is the active tobacco use within 14 days before surgery. UEBMI, Beijing Urban Employee Basic Medical Insurance; BMI, body mass index (kg/m²), categorized based on Chinese adult standards. Chest tube size was measured based on the standard internal diameter of the tube in French units. Comparisons between groups were performed using Welch’s t-test for continuous variables and Pearson’s chi-square test for categorical variables. Boldface indicates statistical significance (***p ≤ 0.001, **p ≤ 0.01, *p ≤ 0.05).

### 3.2 Overlap-weighted univariate logistic regression models

After applying overlap weighting, covariate balance was substantially improved (SMDs < 0.1; **Supplementary Figure 1**), and the weighted propensity score distributions showed substantial overlap between the two groups (**Supplementary Figure 2**). The adjusted effective sample sizes were 151.6 for the routine group and 856.9 for the modified group, with an overall effective sample size of 515.3, supporting adequate overlap and stable weights.

In univariate logistic regression models, the modified placement was associated with lower odds of acute postoperative pain compared with the routine method (odds ratio [OR] = 0.54, 95% CI: 0.36-0.81, *p* = 0.003). None of the demographic factors (age group, sex, education) or smoking history showed significant associations after weighting, Preoperative NSAIDs use history (OR = 1.23, 95% CI: 1.08-1.41, *p* = 0.002), longer drain duration (OR = 1.18, 95% CI: 1.08-1.29, *p* < 0.001), and extended length of inpatient (OR = 1.14, 95% CI: 1.04-1.24, *p* < 0.001) were associated with increased odds of acute postoperative pain individually (**Table 2**).

**Table 2:** Bivariate overlap-weighted logistic regression analysis of the association between independent variables with acute postoperative pain scores (n= 1,082).

Variables	OR	95% CI	*p*-value
ICT placement			
Routine	Ref		
Modified	**0.54**	**[0.36, 0.81]**	0.003^**^
Age			
< 65	Ref		
≥65	1.06	[0.66, 1.70]	0.797
Biological sex			
Female	Ref		
Male	0.79	[0.50, 1.23]	0.292
BMI (kg/m²)			
18.5-24.0	Ref		
24.0-28.0	0.75	[0.42, 1.33]	0.326
≥28.0	1.42	[0.78, 2.60]	0.249
Highest education level			
High school or lower	Ref		
College or higher	0.99	[0.61, 1.61]	0.980
Insurance status			
UEBMI	Ref		
Others	1.11	[0.72, 1.74]	0.631
Smoking history			
No	Ref		
Yes	0.88	[0.54, 1.43]	0.598
Chest tube size			
#18	Ref		
#20	0.70	[0.40, 1.22]	0.211
#24	0.59	[0.29, 1.19]	0.139
NSAIDs use history (days)	**1.23**	**[1.08, 1.41]**	0.002^**^
Tumor size (mm)	1.02	[0.99, 1.05]	0.298
Drain duration (days)	**1.18**	**[1.08, 1.29]**	<.001^***^
Operation length (hours)	1.00	[1.00, 1.00]	0.766
Length of inpatient (days)	**1.14**	**[1.04, 1.24]**	0.004^**^

OR = odds ratio. 95% CI = 95% confidence intervals. Ref = reference level. ICT = intercostal chest tube placement. FEV1 = forced expiratory volume in 1 second measured by liters. NSAIDs = Nonsteroidal Anti-Inflammatory Drugs. Smoking history defined as the active tobacco use within 14 days before surgery. UEBMI = Beijing Urban Employee Basic Medical Insurance. BMI = body mass index kg/m², categorized based on Chinese adult standards. Chest tube size was measured based on the standard internal diameter of the tube in French units. Boldface indicates statistical significance ***p ≤ 0.001, **p ≤ 0.01, *p ≤ 0.05.

### 3.3 Overlap-weighted multivariable logistic regression model

The overlap-weighted multivariable model adjusting for baseline covariates indicated that the modified chest tube placement was associated with 48% lower odds of acute postoperative pain compared to the routine method (adjusted OR [aOR] = 0.52, 95% CI 0.35-0.79, *p* = 0.002) (**Table 3**).

**Table 3:** Multivariable overlap-weighted logistic regression model adjusted for baseline covariates (n= 1,082).

Variables	aOR	95% CI	*p*-value
ICT placement			
Routine	Ref		
Modified	**0.52**	**[0.35, 0.79]**	0.002^**^
Age			
< 65	Ref		
≥65	0.95	[0.58, 1.54]	0.830
Biological sex			
Female	Ref		
Male	0.69	[0.38, 1.28]	0.244
BMI			
18.5-24.0	Ref		
24.0-28.0	0.72	[0.39, 1.33]	0.296
≥28.0	1.59	[0.88, 2.89]	0.125
Highest education level			
High school or lower	Ref		
College or higher	1.06	[0.65, 1.73]	0.822
Insurance status			
UEBMI	Ref		
Others	1.08	[0.69, 1.68]	0.736
Smoking history			
No	Ref		
Yes	1.00	[0.51, 1.94]	0.998
Chest tube size			
#18	Ref		
#20	0.70	[0.40, 1.23]	0.216
#24	0.62	[0.30, 1.29]	0.200
NSAIDs use history (days)	**1.26**	**[1.11, 1.43]**	<.001^***^
Tumor size (mm)	1.01	[0.98, 1.04]	0.550

aOR = adjusted odds ratio. 95% CI = 95% confidence intervals. Ref = reference level. ICT = intercostal chest tube placement. FEV_1_ = forced expiratory volume in 1 second measured by liters. NSAIDs = Nonsteroidal Anti-Inflammatory Drugs. Smoking history is the active tobacco use within 14 days before surgery. UEBMI = Beijing Urban Employee Basic Medical Insurance. BMI = body mass index (kg/m^2^), categorized based on Chinese adult standards. Boldface indicates statistical significance (***: *p* ≤ 0.001, **: *p* ≤ 0.01, *: *p* ≤ 0.05).

### 3.4 Pragmatic care-adjusted sensitivity analysis

The pragmatic care-adjusted model incorporated both baseline covariates and postoperative covariates (drain duration, operative time, and length of inpatient) yielded consistent results. The association between the modified technique and lower odds of pain remained statistically significant (aOR = 0.52, 95% CI: 0.34-0.78, *p* = 0.002) (**Table 4**).

**Table 4:** Multivariable overlap weighted logistic regression model adjusted for baseline and postoperative covariates (n= 1,082).

Variables	OR	95% CI	p-value
ICT placement			
Routine	Ref		
Modified	**0.52**	**[0.34, 0.78]**	0.002^**^
Age			
< 65	Ref		
≥65	0.97	[0.59, 1.59]	0.902
Biological sex			
Female	Ref		
Male	0.66	[0.35, 1.25]	0.199
BMI			
18.5-24.0	Ref		
24.0-28.0	0.73	[0.39, 1.36]	0.328
≥28.0	1.51	[0.82, 2.80]	0.185
Highest education level			
High school or lower	Ref		
College or higher	1.00	[0.61, 1.64]	0.993
Insurance status			
UEBMI	Ref		
Others	1.11	[0.71, 1.74]	0.655
Smoking history			
No	Ref		
Yes	1.11	[0.56, 2.22]	0.763
Chest tube size			
#18	Ref		
#20	0.69	[0.39, 1.21]	0.190
#24	0.57	[0.27, 1.18]	0.127
NSAIDs use history (days)	0.98	[0.74, 1.29]	0.888
Tumor size (mm)	1.01	[0.98, 1.04]	0.676
Drain duration (days)	**1.53**	**[1.08, 2.16]**	0.016^*^
Operation length (hours)	0.91	[0.69, 1.20]	0.493
Length of inpatient (days)	0.79	[0.60, 1.03]	0.080

aOR, adjusted odds ratio; 95% CI, 95% confidence intervals; Ref, reference level; ICT, intercostal chest tube placement; FEV_1_, forced expiratory volume in 1 second measured by liters; NSAIDs, Nonsteroidal Anti-Inflammatory Drugs. Smoking history is the active tobacco use within 14 days before surgery. UEBMI, Beijing Urban Employee Basic Medical Insurance; BMI, body mass index (kg/m²), categorized based on Chinese adult standards. Chest tube size was measured based on the standard internal diameter of the tube in French units. Boldface indicates statistical significance (***p ≤ 0.001, **p ≤ 0.01, *p ≤ 0.05).

### 3.5 Absolute and relative risk estimates

Overlap-weighted analyses predicted absolute risk of acute postoperative pain of 27.1% (95% CI: 20.1%-34.2%, *p* < 0.001) for routine placement and 16.8% (95% CI: 14.3%-19.3%, *p* < 0.001) for modified placement group, corresponding to an risk difference (RD) of 10.3% points (95% CI: 2.8%-17.8%; *p* = 0.007) and 38% lower risk of acute postoperative pain for patients who received the modified placement (risk ratio [RR] = 0.62, 95% CI: 0.43-0.81; *p* = 0.001). For clinical interpretability, the RD translates to a derived NNT (number needed to treat) of 10 (95% CI: 6-36), value rounded to whole numbers (**Table 5**; **Supplementary Figure 3**). This indicates that, for this cohort, about one fewer patient with acute postoperative pain was observed for every 10 patients in the modified placement group compared with the routine placement group.

**Table 5:** Overlap-weighted estimates of absolute risk, risk difference, and risk ratio for acute postoperative pain intensity.

Measure	Estimate	95% CI	p-value
Risk of routine tube placement	27.1%	[20.1, 34.2]	–
Risk of modified tube placement	16.8%	[14.3, 19.3]	–
Risk difference (routine-modified)	10.3%	[2.8, 17.8]	0.007^**^
Risk ratio	0.62	[0.43, 0.81]	<.001^***^
NNT	10	[6, 36]	–

All estimates were calculated from overlap-weighted binomial generalized linear models using identity and log link functions to obtain the risk difference and risk ratio within the pseudo-population representing the average treatment effect in the Overlap (ATO) population. Absolute risks are presented descriptively with 95% confidence intervals. *P*-values are reported only for between-group comparisons: for risk difference, H_0_: RD = 0; for risk ratio, H_0_: RR = 1. Number needed to treat (NNT) was calculated as 1/RD, with values were rounded to whole numbers. (***p ≤ 0.001, **p ≤ 0.01, *p ≤ 0.05).

Detailed findings from unweighted bivariate logistic regression models, the multivariable logistic regression model adjusted for baseline covariates, and the multivariable logistic regression model adjusted for both baseline and postoperative covariates are provided in the **Supplementary Tables 1–3.**

## 4 Discussion

### 4.1 Clinical and methodological implications of this modified chest tube trajectory

In this retrospective cohort of 1,082 patients undergoing uniportal VATS lobectomy, the modified tunneled intercostal chest tube placement technique significantly reduced the acute postoperative pain, suggesting that introducing this minor technical refinement to alter the tube trajectory could meaningfully improve early postoperative comfort in the minimally invasive thoracic surgery.

Chest-tube placement and management are repeatedly implicated as dominant sources of discomfort after thoracic surgery. Pain after thoracic surgery is multifactorial, reflecting incision trauma, intercostal nerve irritation, pleural inflammation, and tube-related mechanical strain (1, 31, 32). Large VATS cohorts also affirm that tube number and trajectory are key risk factors for acute postoperative pain, even in minimally invasive settings (33).

The findings of the current study extend the existing literature in several ways. First, rather than focusing on device substitution (e.g., smaller-diameter drains, digital drainage devices), the present study introduced a technical modification to the chest tube insertion trajectory. This technique is immediately implementable in clinical settings, requiring no additional hardware and is cost-neutral. While device-focused studies report reduced pain and improved comfort after VATS, technique refinement that minimizing neurovascular contact offers a complementary and logistically simple strategy for enhancing patient outcomes.

Second, unlike many historical comparisons, we used overlap weighting to mitigate confounding and stabilize variance in the region of clinical equipoise, which improves upon traditional matching or inverse probability weighting approaches that may suffer from extreme weights or residual imbalance. Methodologically, few prior thoracic surgery studies have employed overlap weighting or similar causal-inference techniques. The present study demonstrates its practical application and, together with sensitivity analyses incorporating postoperative covariates, provides a structured analytic approach that may inform future investigations.

Furthermore, our findings reinforce the principle that surgical technique itself is a key determinant of patient-reported outcomes, even in the era of multimodal analgesia and ERAS protocols. While anesthetic and pharmacologic innovations continue to evolve, attention to anatomic preservation and minimal tissue trauma at the procedural level remains fundamental to postoperative comfort and functional recovery. Beyond thoracic surgery, our findings resonate with a broader surgical literature emphasizing that even subtle technical refinements can produce tangible improvements in patient-reported outcomes. Analogous “tunneling” or “protected insertion” strategies have been incorporated into other surgical procedures to enhance patient comfort (34). Collectively, these studies highlight that optimizing tissue handling and neural preservation, rather than relying solely on pharmacologic analgesia or device upgrades remains an important yet often underrecognized approach for enhancing recovery.

### 4.2 Possible mechanisms

The observed reduction in acute postoperative pain with the modified technique likely reflects a combination of neuroanatomical, biomechanical, and inflammatory mechanisms.

From the perspective of neuroanatomical protection, the reduction in acute pain may be attributed to the modified technique safeguarding the intercostal nerves from mechanical injury. Routine direct insertion places the tube adjacent to the inferior border of the rib where intercostal muscle fibers have been divided or cauterized, leaving local tissue laxity and loss of tension after closure (35). As a result, the tube lies in close proximity to the intercostal neurovascular bundle and moves with each breath, cough, or change in posture, repeatedly stimulating the nerve and triggering sharp, radiating chest wall pain (36–38).

By redirecting the tube along the superior border of the lower rib through a short subcutaneous and intramuscular tunnel, the modified approach reduces direct contact with the neurovascular bundle and preserves intact intercostal muscle and fascia around the entry point. This intact tissue functions as a separating layer that likely reduces nociceptor activation and secondary neuropathic sensitization from repetitive micro-motion. In simple terms, the tunnel serves as a soft, biologic guard that buffers the intercostal nerve from pressure and shear forces during normal breathing, coughing, and ambulation.

In addition, we hypothesize that biomechanical stabilization plays a key role in the observed reduction of acute pain. Chest tubes experience cyclic axial traction and lateral shear with respiration, coughing, and patient repositioning. This repetitive movement can generate micro−motion at the skin–tube interface and within the intercostal space, which could contribute to the postoperative discomfort and tissue irritation. The short subcutaneous tunnel of this modified technique creates a composite sleeve of muscle, fascia, and subcutaneous tissue around the tube. This sleeve increases static friction, dampens piston-like axial sliding, improves lateral stability, and distributes forces over a longer tract. The separation of the tube from the main working incision also allows external forces at the incision to dissipate in the subcutaneous segment before reaching the intercostal space, potentially attenuate amplification of pain signals. Future biomechanical and imaging studies are needed to validate these hypotheses and to quantify how tunnel geometry and tissue dynamics contribute to postoperative comfort.

From the perspective of inflammation and sensory modulation, another possible explanation is that reduced mechanical stimulation may help attenuate local inflammatory responses and lower sensory hypersensitivity, thereby contributing to improved acute postoperative pain. Repeated mechanical irritation can amplify local inflammation and peripheral sensitization at the intercostal space. Reduced friction and pressure may potentially help suppress this inflammatory cascade, thereby alleviating nociceptive pain and mitigating the neuropathic pain component that may persist even after minimally invasive operations. Although trials directly comparing “tunnel” versus “no tunnel” techniques are limited, the proposed mechanistic direction aligns with broader perioperative pain evidence in thoracic surgery where approaches that protect intercostal nerves or reduce local irritation are associated with lower pain scores or analgesic needs (39, 40).

Furthermore, based on our operative experience, a tunnel length of approximately 2 cm within the same intercostal space provides an optimal balance between tube stability and drainage patency. If the tunnel length is less than 1 cm, the surrounding muscle and fascial support may be inadequate, leading to greater tube sliding and potentially increasing frictional pain. Conversely, if the length exceeds approximately 3 cm, the curvature of the subcutaneous tract may become excessive, raising the risk of kinking, localized traction, or impaired drainage. Thus, this 2 cm tunnel length can be framed as a pragmatic balance informed by anatomical considerations and the need to minimize bending radius while ensuring adequate sleeve support, although the exact length may vary slightly depending on patient anatomy. Future comparative studies that randomize or standardize tunnel length are warranted to validate or refine this tunnel length.

## 5 Study limitations

The findings of this study should be interpreted in light of limitations. First, the retrospective cohort design inherently limits causal inference. Although overlap weighted multivariable logistic regression models were applied to adjust for baseline confounding, residual and unmeasured confounding cannot be completely excluded. Other potential confounding factors such as intraoperative analgesic dosing, surgical expertise, and patient personal pain thresholds may still have influenced the outcomes but were not available in this derived EHR data. In addition, given the large sample accumulated over two years, time-related effects may exist in clinical practice and could contribute to residual confounding despite adjustment for measured covariates. Second, postoperative acute pain assessment in this study was limited to resting pain scores routinely collected in clinical practice. Other dimensions of pain, such as pain during coughing, ambulation (e.g. slow walking, changing position in bed), or deep breathing, were not systematically evaluated (23). This limited assessment may underestimate the overall burden of pain and the functional advantages that the modified tunneled intercostal insertion technique might confer. Third, the study relied on data documented in EHR, which may be subject to reporting variability across time and nursing staff, which could potentially affect the comparability of pain scores across different patients. Fourth, several functional recovery endpoints that are relevant to evaluate ERAS-related benefits, including standardized postoperative opioid consumption (41), time to first ambulation, and consistently recorded pulmonary complications, were not available in this EHR-based dataset. Accordingly, our findings should be interpreted as primarily reflecting postoperative acute resting pain rather than broader recovery outcomes. Fifth, patients who developed severe postoperative complications (Clavien-Dindo grade III-V) were excluded from the current analysis as these events are associated with prolonged drainage, analgesic escalation, and altered recovery pathways. This exclusion could limit generalizability to patients with complicated postoperative courses. Sixth, this study was conducted at a single tertiary hospital. Although the site is one of the largest medical centers in the region and serves a densely populated metropolitan area with more than 20 million residents, this may still limit the generalizability of the findings to other institutions with different postoperative care protocols or distinct patient demographics. Future multicenter prospective studies are warranted to validate these results across broader clinical settings. Finally, this study focused on acute postoperative pain and did not evaluate longer-term outcomes such as chronic pain, functional recovery, or quality of life after the surgery. Future studies incorporating standardized, multidimensional pain assessments, structured analgesic utilization metrics, functional recovery endpoints, and long-term follow-up are warranted to better understand the comprehensive effects of this aforementioned modified chest tube placement techniques after VATS. Nonetheless, the findings from the current study provide meaningful insights for the continued exploration and optimization of chest tube placement techniques aimed at reducing the acute postoperative pain levels and improving patient comforts.

## 6 Conclusion

Although further multicenter prospective randomized controlled trials are needed to valid these findings and establish causality, the findings indicate that the tunneled intercostal chest tube placement technique could serve as a simple, low-cost, and easily implementable modification to improve acute postoperative pain following uniportal VATS. Since the technique does not require additional instruments, advanced training, or extended operative time, it could be readily integrated into standard chest tube insertion protocols without increasing procedural complexity or cost. Reducing acute pain after thoracic procedures is critical not only for patient satisfaction but also for facilitating early ambulation, supporting pulmonary rehabilitation, and minimizing the use of opioids. Integrating this feasible modified chest tube placement technique into standard postoperative protocols may therefore contribute to faster recovery and improved overall patient outcomes.

## Data Availability

The raw data supporting the conclusions of this article will be made available by the authors, without undue reservation.
