# Supplementary material for: Association between modified same-intercostal chest tube placement and acute postoperative pain after uniportal VATS lobectomy: an overlap weighted retrospective cohort study
Source: Front Oncol. 2026 Apr 13;16:1797512. doi: 10.3389/fonc.2026.1797512 (PMC13110948; doi:10.3389/fonc.2026.1797512)
Supplement: Supplementary file 1 [file DataSheet1.docx]

***Supplementary Material***

**Table 1.** Unweighted bivariate logistic regression analysis of the association between independent variables with acute postoperative pain scores (n= 1,082)

**Table 2.** Unweighted multivariable logistic regression model adjusted for baseline covariates (n= 1,082)

**Table 3.** Unweighted multivariable logistic regression model adjusted for baseline and postoperative covariates (n= 1,082)

**Figure 1.** Covariate balance plot (LOVE) before and after overlap weighting

**Figure 2.** Propensity score distributions density plot after overlap weighting

**Figure 3.** Marginal predicted risks of acute postoperative pain

**Supplementary Material 1.** Recommended standard operating procedures

**Supplementary Table 1.** Unweighted bivariate logistic regression analysis of the associations between independent variables with acute postoperative pain intensity (n= 1,082)

| Variables | OR | 95% CI | *p*-value |
| --- | --- | --- | --- |
| ICT placement |  |  |  |
| Routine | Ref |  |  |
| Modified | **0.56** | **[0.38, 0.84]** | **0.004^**^** |
| Sociodemographic |  |  |  |
| Age |  |  |  |
| < 65 | Ref |  |  |
| ≥65 | 0.98 | [0.71, 1.34] | 0.889 |
| Biological sex |  |  |  |
| Female | Ref |  |  |
| Male | 0.88 | [0.65, 1.20] | 0.430 |
| BMI (kg/m^2^) |  |  |  |
| [18.5-24.0) | Ref |  |  |
| [24.0-28.0) | 1.10 | [0.75, 1.60] | 0.607 |
| ≥28.0 | 1.38 | [0.88, 2.11] | 0.146 |
| Highest education level |  |  |  |
| High school or lower | Ref |  |  |
| College or higher | 1.00 | [0.71, 1.40] | 0.980 |
| Insurance status |  |  |  |
| UEBMI | Ref |  |  |
| Others | 1.02 | [0.75, 1.40] | 0.892 |
| Smoking history |  |  |  |
| No | Ref |  |  |
| Yes | 1.09 | [0.78, 1.51] | 0.610 |
| Chest tube size |  |  |  |
| #18 | Ref |  |  |
| #20 | 0.86 | [0.59, 1.28] | 0.454 |
| #24 | 0.85 | [0.52, 1.40] | 0.532 |
| NSAIDs use history (days) | **1.19** | **[1.09, 1.29]** | **<.001^***^** |
| Tumor size (mm) | 0.99 | [0.97, 1.01] | 0.437 |
| Drain duration (days) | **1.14** | **[1.08, 1.20]** | **<.001^***^** |
| Operation length (hours) | 1.00 | [1.00, 1.00] | 0.294 |
| Length of inpatient (days) | **1.09** | **[1.04, 1.15]** | **<.001^***^** |

*Notes:* OR = odds ratio. 95% CI = 95% confidence intervals. Ref = reference level. ICT = intercostal chest tube placement. FEV_1_ = forced expiratory volume in 1 second measured by liters. NSAIDs = Nonsteroidal Anti-Inflammatory Drugs. Smoking history is the active tobacco use within 14 days before surgery. UEBMI = Beijing Urban Employee Basic Medical Insurance. BMI = body mass index (kg/m²), categorized based on Chinese adult standards. Chest tube size was measured based on the standard internal diameter of the tube in French units. Boldface indicates statistical significance (***: *p* ≤ 0.001, **: *p* ≤ 0.01, *: *p* ≤ 0.05).

**Supplementary Table 2.** Unweighted multivariable logistic regression model adjusted for baseline covariates (n= 1,082)

| Variables | aOR | 95% CI | *p*-value |
| --- | --- | --- | --- |
| ICT placement |  |  |  |
| Routine | Ref |  |  |
| Modified | **0.53** | **[0.36, 0.81]** | **0.003^**^** |
| Sociodemographic |  |  |  |
| Age |  |  |  |
| < 65 | Ref |  |  |
| ≥65 | 0.93 | [0.66, 1.32] | 0.703 |
| Biological sex |  |  |  |
| Female | Ref |  |  |
| Male | 0.67 | [0.43, 1.04] | 0.078 |
| BMI (kg/m^2^) |  |  |  |
| [18.5-24.0) | Ref |  |  |
| [24.0-28.0) | 1.13 | [0.76, 1.65] | 0.539 |
| ≥28.0 | 1.40 | [0.89, 2.17] | 0.136 |
| Highest education level |  |  |  |
| High school or lower | Ref |  |  |
| College or higher | 1.02 | [0.70, 1.46] | 0.918 |
| Insurance status |  |  |  |
| UEBMI | Ref |  |  |
| Others | 1.05 | [0.75, 1.45] | 0.793 |
| Smoking history |  |  |  |
| No | Ref |  |  |
| Yes | 1.42 | [0.89, 2.28] | 0.149 |
| Chest tube size |  |  |  |
| #18 | Ref |  |  |
| #20 | 0.86 | [0.58, 1.29] | 0.455 |
| #24 | 0.85 | [0.51, 1.40] | 0.519 |
| NSAIDs use history (days) | **1.21** | **[1.11, 1.32]** | **<.001^***^** |
| Tumor size (mm) | 0.99 | [0.97, 1.01] | 0.269 |

*Notes:* aOR = adjusted odds ratio. 95% CI = 95% confidence intervals. Ref = reference level. ICT = intercostal chest tube placement. FEV_1_ = forced expiratory volume in 1 second measured by liters. NSAIDs = Nonsteroidal Anti-Inflammatory Drugs. Smoking history is the active tobacco use within 14 days before surgery. UEBMI = Beijing Urban Employee Basic Medical Insurance. BMI = body mass index (kg/m²), categorized based on Chinese adult standards. Chest tube size was measured based on the standard internal diameter of the tube in French units. Boldface indicates statistical significance (***: *p* ≤ 0.001, **: *p* ≤ 0.01, *: *p* ≤ 0.05).

**Supplementary Table 3.** Unweighted multivariable logistic regression model adjusted for baseline and postoperative covariates (n= 1,082)

| Variables | aOR | 95% CI | *p*-value |
| --- | --- | --- | --- |
| ICT placement |  |  |  |
| Routine | Ref |  |  |
| Modified | **0.53** | **[0.35, 0.82]** | **0.003^**^** |
| Sociodemographic |  |  |  |
| Age |  |  |  |
| < 65 | Ref |  |  |
| ≥65 | 0.95 | [0.67, 1.35] | 0.787 |
| Biological sex |  |  |  |
| Female | Ref |  |  |
| Male | 0.66 | [0.41, 1.02] | 0.069 |
| BMI (kg/m^2^) |  |  |  |
| 18.5-24.0 | Ref |  |  |
| 24.0-28.0 | 1.16 | [0.77, 1.71] | 0.469 |
| ≥28.0 | 1.39 | [0.87, 2.18] | 0.162 |
| Highest education level |  |  |  |
| High school or lower | Ref |  |  |
| College or higher | 0.98 | [0.67, 1.43] | 0.932 |
| Insurance status |  |  |  |
| UEBMI | Ref |  |  |
| Others | 1.03 | [0.74, 1.45] | 0.856 |
| Smoking history |  |  |  |
| No | Ref |  |  |
| Yes | 1.47 | [0.91, 2.41] | 0.121 |
| Chest tube size |  |  |  |
| #18 | Ref |  |  |
| #20 | 0.84 | [0.56, 1.28] | 0.405 |
| #24 | 0.82 | [0.49, 1.37] | 0.450 |
| NSAIDs use history (days) | 0.96 | [0.79, 1.17] | 0.696 |
| Tumor size (mm) | 0.98 | [0.96, 1.01] | 0.200 |
| Drain duration (days) | **1.35** | **[1.09, 1.71]** | **0.009^**^** |
| Operation length (hours) | 1.03 | [0.84, 1.25] | 0.772 |
| Length of inpatient (days) | 0.87 | [0.73, 1.02] | 0.109 |

*Notes:* aOR = adjusted odds ratio. 95% CI = 95% confidence intervals. Ref = reference level. ICT = intercostal chest tube placement. FEV_1_ = forced expiratory volume in 1 second measured by liters. NSAIDs = Nonsteroidal Anti-Inflammatory Drugs. Smoking history is the active tobacco use within 14 days before surgery. UEBMI = Beijing Urban Employee Basic Medical Insurance. BMI = body mass index (kg/m²), categorized based on Chinese adult standards. Chest tube size was measured based on the standard internal diameter of the tube in French units. Boldface indicates statistical significance (***: *p* ≤ 0.001, **: *p* ≤ 0.01, *: *p* ≤ 0.05).


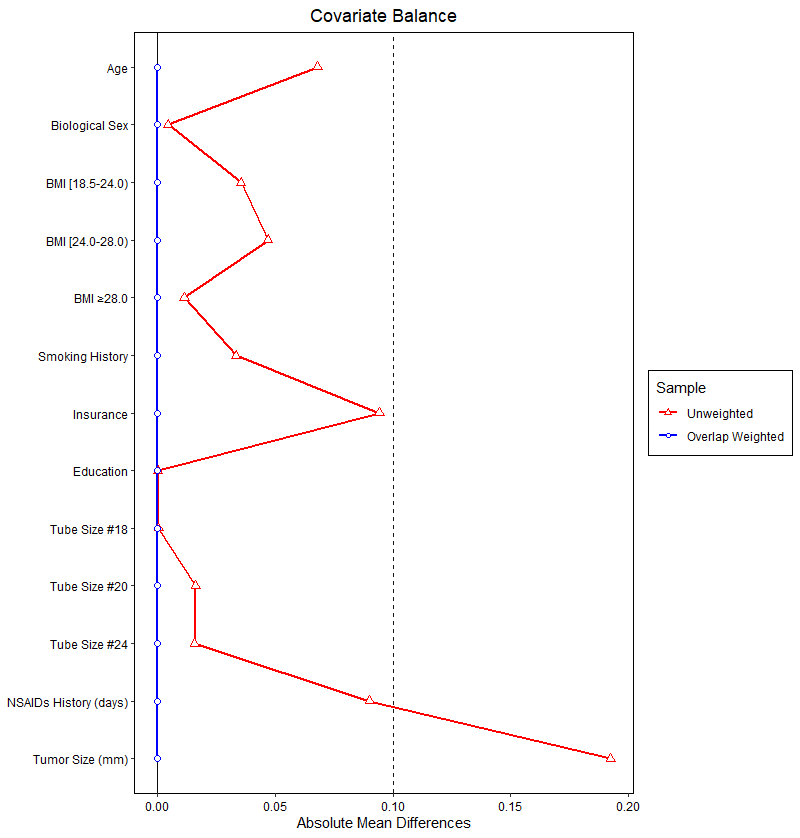


**Supplementary Figure 1.** Covariate balance plot (LOVE) before and after overlap weighting**.** Standardized mean differences (SMDs) of baseline covariates before and after overlap weighting. The dotted vertical line at |SMD| = 0.1 indicates the threshold for acceptable balance. Overlap weighting substantially improved covariate balance across all variables between the modified and routine chest tube placement groups.


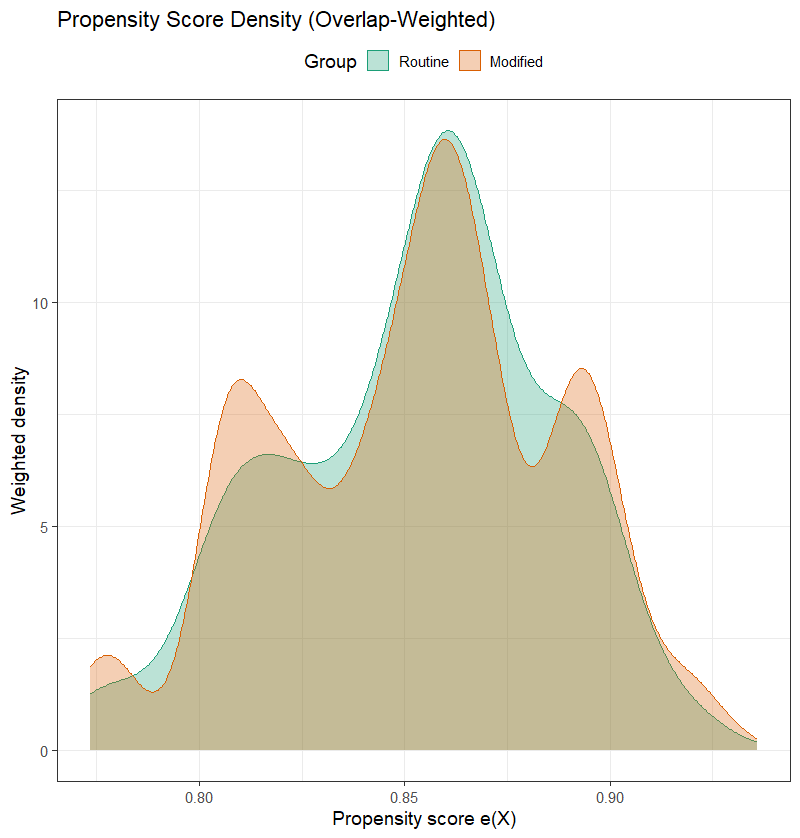


**Supplementary Figure 2.** Propensity score distributions density plot after overlap weighting. Density plots of the estimated propensity score distributions for the modified and routine chest tube placement groups after overlap weighting. Weighting leads to substantial overlap of the two distributions, indicating improved comparability between groups.


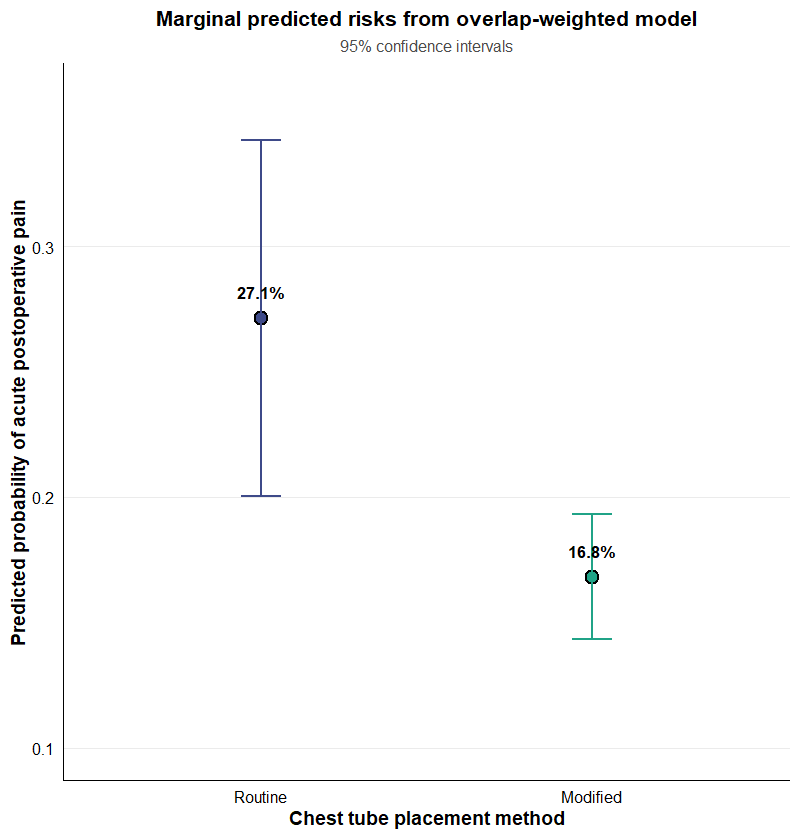


**Supplementary Figure 3.** Marginal predicted probabilities of acute postoperative pain after overlap weighting. Points represent overlap weight-adjusted absolute risks, and vertical bars indicate 95% confidence intervals.

**Supplementary Material 1. Recommended standard operating procedures**

**Disclaimer:** The following SOP describes the modified tunneled chest tube placement approach used in this study. It is presented solely to document the procedural steps for research reporting and should not be interpreted as a substitute for formal surgical training, institutional credentialing, or professional clinical judgement. Clinicians should follow applicable local policies and standards of care and adapt steps as clinically appropriate.

**Modified tunneled chest tube placement after uniportal VATS lobectomy**

The purpose of this modified technique is to create a short subcutaneous tunnel within the same intercostal space as the uniprotal incision and insert the chest tube through the intercostal muscle along the superior border of the lower rib.

**Step 1.** Identify the exit point

Before skin closure, identify the chest tube exit point at the posterior margin of the uniportal incision, approximately 2 cm posterior to the wound edge, within the same intercostal space. No additional skin incision is created.

**Step 2.** Establish the dissection plane

Confirm the intended tunnelling plane is the subcutaneous tissue plane above the intercostal muscle layer, with the tunnel direction oriented parallel to the intercostal space.

**Step 3.** Create the subcutaneous tunnel

Using curved hemostatic forceps, perform blunt dissection only to create a short subcutaneous tunnel. Advance the forceps gently posteriorly in parallel with the intercostal space to form a tunnel of approximately 2 cm in length. Avoid sharp dissection.

**Step 4.** Control depth and protect the neurovascular bundle

Limit dissection depth to the intercostal muscle layer overlying the superior border of the lower rib. Avoid the inferior margin of the upper rib to reduce risk of injury to the intercostal neurovascular bundle.

**Step 5.** Pleural entry and tube insertion under thoracoscopic visualization

At the posterior end of the tunnel, enter the pleural cavity by passing perpendicularly through the intact intercostal muscle layer along the superior border of the lower rib. Advance the chest tube through the tunnel until its tip emerges from the posterior exit point and enters the pleural cavity. Adjust tube position under thoracoscopic visualization so the distal tip is directed toward the lung apex or posterior basal region. Excessive bleeding is uncommon during this step because the approach follows separation through the intercostal tissues along an anatomic plane. Hemostasis, if required, is managed using standard intraoperative measures.

**Step 6.** Fixation, dressing, and verification

Secure the chest tube to the skin using a non-absorbable suture, consistent with the standard fixation method used in both the routine and modified techniques. Apply dressing according to the institution’s standard practice (same as routine technique). Obtain a routine chest X-ray within 24 hours after surgery to confirm tube position and lung re-expansion.
